# Supplementary material for: MetaRibo-Seq measures translation in microbiomes
Source: Nat Commun. 2020 Jun 29;11:3268. doi: 10.1038/s41467-020-17081-z (PMC7324362; doi:10.1038/s41467-020-17081-z)
Supplement: Supplementary file 10 — Supplementary Data 7 [file 41467_2020_17081_MOESM10_ESM.zip › File2/Confidence_High_Taxonomy/156603_out.krona.html]

Javascript must be enabled to view this page.

members
magnitude
magnitudeUnassigned
count
unassigned
taxon
rank

156603\_out

13

superkingdom
13
2

phylum
13
1239

13
class
186801

13
order
186802

541000
family
12

1263
12
genus

12
species
46228

SRS013098\_contig\_number\_40591SRS015890\_contig\_number\_25412SRS017916\_contig\_number\_15681SRS019601\_contig\_number\_3856SRS019685\_contig\_number\_40370SRS048164\_contig\_number\_14785SRS051031\_contig\_number\_14206SRS062701\_contig\_number\_6343SRS098571\_contig\_number\_12756SRS143181\_contig\_number\_2027SRS147766\_contig\_number\_2463SRS148784\_contig\_number\_contig-100\_21966.60334

1
family
186803

1
genus
841


SRS015663\_contig\_number\_17504
360807
species
1
